# Transcriptomic Reprogramming and Genetic Variations Contribute to Western Hemlock Defense and Resistance Against Annosus Root and Butt Rot Disease

**DOI:** 10.3389/fpls.2022.908680

**Published:** 2022-06-30

**Authors:** Jun-Jun Liu, Arezoo Zamany, Charlie Cartwright, Yu Xiang, Simon F. Shamoun, Benjamin Rancourt

**Affiliations:** ^1^Natural Resources Canada, Canadian Forest Service, Victoria, BC, Canada; ^2^British Columbia Ministry of Forests, Cowichan Lake Research Station, Mesachie Lake, BC, Canada; ^3^Summerland Research and Development Centre, Agriculture and Agri-Food Canada, Summerland, BC, Canada

**Keywords:** Annosus root and butt rot disease, de novo assembly, quantitative resistance, transcriptome profiling, western hemlock

## Abstract

Western hemlock (*Tsuga heterophylla*) is highly susceptible to Annosus root and butt rot disease, caused by *Heterobasidion occidentale* across its native range in western North America. Understanding molecular mechanisms of tree defense and dissecting genetic components underlying disease resistance will facilitate forest breeding and disease control management. The aim of this study was to profile host transcriptome reprogramming in response to pathogen infection using RNA-seq analysis. Inoculated seedlings were clearly grouped into three types: quantitative resistant (QR), susceptible (Sus), and un-infected (Uif), based on profiles of *H. occidentale* genes expressed in host tissues. Following *de novo* assembly of a western hemlock reference transcriptome with more than 33,000 expressed genes, the defensive transcriptome reprogramming was characterized and a set of differentially expressed genes (DEGs) were identified with gene ontology (GO) annotation. The QR seedlings showed controlled and coordinated molecular defenses against biotic stressors with enhanced biosynthesis of terpenoids, cinnamic acids, and other secondary metabolites. The Sus seedlings showed defense responses to abiotic stimuli with a few biological processes enhanced (such as DNA replication and cell wall organization), while others were suppressed (such as killing of cells of other organism). Furthermore, non-synonymous single nucleotide polymorphisms (ns-SNPs) of the defense- and resistance-related genes were characterized with high genetic variability. Both phylogenetic analysis and principal coordinate analysis (PCoA) revealed distinct evolutionary distances among the samples. The QR and Sus seedlings were well separated and grouped into different phylogenetic clades. This study provides initial insight into molecular defense and genetic components of western hemlock resistance against the Annosus root and butt rot disease. Identification of a large number of genes and their DNA variations with annotated functions in plant resistance and defense promotes the development of genomics-based breeding strategies for improved western hemlock resistance to *H. occidentale.*

## Introduction

Western hemlock (*Tsuga heterophylla* Rafinesque) is an important conifer species, with natural distribution from southern Alaska along the Pacific coast to northern California, as well as in northern Rocky Mountains from central regions of British Columbia and Alberta, to Montana and Idaho at elevations from 0 to 1,830 m. It usually grows to 30–50 m, up to 70 m tall and 1–1.5 meters in diameter, with considerable values for lumber and paper production (King et al., 1998). In addition, its cambium was a food source for Native Americans (Whitney, 1985) and its bark is rich in tannins and has provided major raw material for leather tanning for over 100 years (Hergert, 1989). Western hemlock has good shade tolerance, resulting in its abundant growth underneath mature trees of other species in the forest and making it an important food source for deer and elk.

Annosus root and butt rot is caused by the *Heterobasidion annosum sensu* lato complex (Garbelotto and Gonthier, 2013) and poses fundamental threats to biodiversity and the forest industry because it infects a large number of forest species (Asiegbu et al., 2005; Otrosina and Garbelotto, 2010). Fungal spores spread above ground *via* wind and invade wounds on stems, or through cut surfaces of fresh stumps. Below ground root contact is the main pathway to spread pathogen from the infected stumps to nearby healthy trees (Garbelotto and Gonthier, 2013). After infection, young trees usually show disease symptoms typical of other root diseases, such as chlorotic foliage, reduction in leader and branch growth, and a distressed cone crop. Although external symptoms of more mature infected trees are not easy discernible, they develop butt rot resulting in wood decay. Trees with infected roots gradually die, or weaken, and are blow down in strong winds over time, creating mortality pockets in the forest, and are visible from aerial views (Demchik et al., 2020). Some disease management options, such as stump removal, stump treatments with fungicide chemicals or microbial solutions, are available for disease controls (Morrison and Johnson, 1999; Cleary et al., 2013), but the costs are high with negative ecological impacts (Moffat et al., 2011), and with limited effects (Oliva et al., 2010; Demchik et al., 2020).

Of the five species in the *H. annosum sensu* lato complex, *H. occidentale* is a native pathogen with a distribution range across western North America. Western hemlock and several other broadleaf species are highly susceptible to *H. occidentale*, causing serious loss to the forest industry (Morrison, 1979; Schmitt et al., 2000). Hemlock trees have shallow root systems, making infected trees more susceptible to wind blow and fire damage (Eis, 1974). It appears that planting trees with disease resistance or tolerance is an ideal strategy for management of forest diseases (Sniezko and Liu, 2022). Western hemlock breeding began in the early 1950s with parent tree selection, testing and selection of seed families for establishment of seed orchards (Jayawickrama, 2003; Cappa et al., 2015; Cartwright and King, 2021). This was a co-operative effort across the forest industry, British Columbia (BC) Ministry of Forest, government agencies and First Nations from Washington and Oregon (King and Cartwright, 1999). These tree improvement programs have aimed at genetic gains with improved growth, but association between improved tree growth and adaptive traits to environmental stressors is uncertain (Hannerz et al., 1999). Although pest resistance was initially assessed at the species level by comparison between western hemlock and other *Tsuga* spp. (Oten et al., 2012), intraspecific variation of western hemlock resistance to pests/pathogens was rarely investigated (Rose et al., 2019). Genetic diversity and related resistance or tolerance to root-rot diseases are still unknown, and disease-resistant stocks await to be developed in western hemlock.

In this study, a composite western hemlock seed family was evaluated for differential defense responses to inoculation by *H. occidentale*. We performed dual RNA-seq of the inoculated seedlings to generate a reference transcriptome for western hemlock and to further reveal the complex molecular host–pathogen interactions and genetic diversity across the samples. The primary objective was to identify defense-responsive and resistance-related genes for future selection of genotypes with enhanced resistance in this conifer species.

## Materials and Methods

### Plant Inoculation

Seeds of western hemlock seedlot Hw #63248 were sowed, and the seedlings were grown at the Cowichan Lake Research Station (Victoria, BC). They were moved to the Pacific Forestry Centre and inoculated in November 2013 at three-years-old. Hw #63248 was composed of seeds pooled from about 15 elite parent trees selected from across the range in BC and Washington State.

*Heterobasidion occidentale* isolate # 5190 was collected by Dr. Brenda Callan (retired mycologist, PFC-CFS) from a diseased western hemlock tree at Ladysmith, BC, Canada on May 31, 2010. It was cultured into pure isolate by several rounds of continuous sub-culture on 2% malt extract and 1.5% agar (MEA), and stored in water agar at 4°C before it was used for inoculation trial. Inoculation was performed following a protocol (Bonello et al., 2008) with a few modification. In brief, *H. occidentale* strain was cultured on MEA plates for about 1 week. Disks of agar culture 5 mm in diameter were taken from the margins of actively growing cultures as inoculum. A plug of bark was removed from each seedling at approximately 5 cm above the root collar at the base of the stem using a cork borer cleaned with 70% ethanol. This punched hole was filled with an agar inoculum disk with the fungal culture facing inward, and sealed by wrapping with parafilm. Mock-inoculated wound controls were included by placing sterile plugs of malt extract agar in the hole of the stem. A total of 90 seedlings were used in two inoculations trials, including 60 inoculated with the fungus, 30 as mock-inoculated wound controls.

Post-inoculation, seedlings were randomized and grown at room temperature in a greenhouse. Growth rates and disease severity were monitored until seedlings were sampled. Morphological traits were firstly used to assess the disease progression post-inoculation, including needle shed, foliage chlorosis, and growth of leader and branch. A subset of western hemlock seedlings with disease symptoms were sampled to check for the presence/absence of dark stain of wood decay in the stem tissues at the inoculation site. Infected seedlings showed staining in the wood, while none of the mock-inoculated seedlings had any visible stain at the wounding sites. Stem wood selections were placed on 2% MEA plates and incubated at room temperature to confirm the presence of the fungus. Cultures from infected seedlings were confirmed as *H. occidentale* based on microscopic diagnosis of the fungus. For metatranscriptomic analysis by RNA-seq, stems sections in ~ 2 cm length, including bark and wood tissues, were collected at the inoculation site and the root collar separately for each seedlings at 22 months post-inoculation. The collected materials were immediately frozen in liquid nitrogen, and stored at –80°C before RNA extraction.

### RNA Extraction and RNA-Seq Analysis

Total RNA extraction was performed as described previously (Liu et al., 1998). After removal of genomic DNA by DNase I digestion, an RNeasy Plant Mini Kit (Qiagen) was used to collect pure total RNA. RNA concentration was determined using NanoDrop 2000 spectrophotometer (Thermo Scientific) and Qubit RNA assay, and its integrity was verified by agarose gel electrophoresis and RNA 6000 Nano assay using Agilent BioAnalyzer 2100. Messenger RNA was purified from total RNA using oligo-dT. Illumina TruSeq mRNA stranded library was prepared using the Illumina TruSeq RNA Library Prep Kit. A total of 30 cDNA libraries were constructed for 15 seedlings, each seedling with two stem sections harvested at inoculation site and root collar separately (Supplementary Table S1). Sequencing was carried out using Illumina HiSeq, which generated a total of 813 million 100-bp PE reads. RNA-seq raw reads were deposited in GenBank (BioProject ID PRJNA791410).

### Detection of *Heterobasidion occidentale* Genes Expressed in Hemlock Stem Tissues

RNA-seq reads were first filtered by Trimmomatic^1^ with default setting at Illuminaclip: TruSeq3-PE.fa:2:30:10, Leading: 3, Trailing: 3, Slidingwindow: 4:15, Minlen: 36 (Bolger et al., 2014). To detect *H. occidentale* genes expressed in Hemlock stem tissues, RNA-seq reads were mapped to a reference transcriptome assembled from RNA-seq reads of *H. occidentale* mycelium cultures in a previous study (Liu et al., 2018). Mapping was performed using CLC Genomics Workbench (v5.5) with minimum length fraction = 0.9, and minimum similarity fraction = 0.98. The numbers of *H. occidentale* genes expressed in host tissues were calculated, and normalized as number of expressed genes per million reads inputted for mapping (NGPM). The ratio of NGPM in the root collar to that in the inoculation site was considered as the best variable to distinguish resistance versus susceptibility at the individual level.

### *De novo* Assembly of a Hemlock Reference Transcriptome

To generate a hemlock reference transcriptome, the clean PE reads were used for *de novo* assembly using Trinity (version: v2.8.5, released on May 24, 2019; Haas et al., 2013). Because the hemlock samples were collected from the greenhouse with soil contamination, the assembled transcripts were filtered by gene-level expression estimates with transcripts per million (TPM) ≥ 1 in at least one of 30 samples using RSEM v1.3.3 (Li and Dewey, 2011). The longest transcript for each gene was picked up as a reference sequence for the gene, and subjected to BLASTx searches (*e*-value < 1*e*–6) against NCBI nr database and proteomes of conifers (sugar pine and white spruce) with draft genome sequences. Metagenome Analyzer, MEGAN6 (Huson et al., 2016), was further used for sequence assignment to putative taxonomical categories: viridiplantae, fungi, bacteria, and others. Following the removal of sequences from potentially other taxonomic origins, a set of transcripts was extracted as a western hemlock reference transcriptome for further analysis.

### Identification of Differentially Expressed Genes (DEGs) in Hemlock Defense in Response to Fungal Infection

DEGs were evaluated between seedling groups with different rates of infection. The clean RNA-seq reads were mapped to the hemlock reference transcriptome using CLC Genomics Workbench v5.5 with parameters as described above except: minimum length fraction (long reads) = 0.90, minimum similarity fraction (long reads) = 0.95. Reads per kilo base per million mapped reads (RPKM) was used to evaluate gene expression levels. RPKM values with proportional fold change ≥ 2 at *p* < 0.05 after adjustment for false discovery rate (FDR) were called to detect DEGs.

A putative proteome was predicted from the *de novo* assembled transcriptome using TransDecoder in the Trinity software package at a minimum protein length of 50. To check the western hemlock proteome, reciprocal BLAST analysis was performed using protein datasets from other conifer species. Gene ontology (GO) analysis was performed using OmicsBox.^2^ GO term enrichment analysis was performed using the Fisher Exact Test method with the whole transcriptome used as a reference and *p* values adjusted by FDR. The GO categories with reduced redundancy were prepared using the REViGO tool (Supek et al., 2011) through its online service.^3^

### Gene Expression Analysis by Reverse Transcription Quantitative Real-Time PCR (RT-qPCR)

Total RNA was used for cDNA synthesis using random hexamers with a SuperScript VILO cDNA synthesis kit (Invitrogen), following the manufacturers’ instructions. SYBR green PCR core reagents (Applied Biosystems) were used to prepare PCR reaction mixtures, and qPCR was run with technical repeats on an Applied Biosystems 7500 Fast Real-time PCR System (Life Technologies). A unique gene product per primer pair was confirmed by melt curve analysis and a distinct peak in the fluorescence vs. temperature analysis of the amplicon. RNA-seq data revealed that the transcript for translation elongation factor EF-1 subunit alpha was evenly expressed across the sample set. This hemlock gene was selected as the reference gene and included in the 96-well plates for RT-qPCR runs. PCR reactions with cDNA synthesis reactions lacking reverse-transcriptase or no template controls were also included as negative controls in qPCR runs for each targeted gene. Relative gene expression levels were calculated as relative quantification (RQ) with 2^−ΔΔCt^ using the Expression Suite software (v1.0.3, Life Technologies).

### Genetic Diversity Analysis Based on SNPs Mined From RNA-Seq Reads

DNA variations were detected by mapping clean RNA-seq reads against a set of defense-related candidate genes extracted from the hemlock reference transcriptome. The defense-related genes were selected based on a gene expression study and GO annotation, including DEGs as revealed above and other genes with GO terms as related to “defense response,” and sequence description as related to “disease resistance protein,” “protein with nucleotide-binding site (NBS) and leucine-rich repeats (LRR) domain (NLR),” “leucine-rich receptor-like protein kinase family protein (LRR-RLK),” “receptor-like protein kinase” (RLK), etc.

The CLC Genomics Workbench (v5.5, Aarhus, Denmark) was used to align RNA-seq reads to reference sequences of the selected genes with parameters: masking mode = no masking; mismatch cost = 2; insertion cost = 3; deletion cost = 3; length fraction = 0.90; similarity fraction = 0.98; auto-detect paired distances = yes; global alignment = yes; non-specific match handling = ignore. DNA variations (SNP, MNV, and InDel) were then called with parameters: minimum coverage = 20; maximum expected variants = 2; ignore quality scores = no; ignore non-specific matches = yes; ignore broken pairs = yes; variant probability = 90.0; require presence in both forward and reverse reads = yes.

To analyze genetic diversity, non-synonymous SNPs (ns-SNPs) were selected for each gene from their putative coding regions. Because ns-SNPs cause amino acid changes with potential for dramatic changes in the biochemical properties of the encoded proteins (for example, changes between neutral and acidic or basic amino acids), thus they may affect the function of candidate genes involved in genetic resistance to pathogenic infection. Ns-SNP loci with no missing data were selected to generate a sequence by concatenating polymorphic loci for each seedling. Following multiple alignment of sequences using Clustal Omega program (Sievers et al., 2011), phylogenetic trees were constructed by using maximum likelihood (ML) analysis method with 100 bootstrap replicates in the programs MEGA X with default options (Kumar et al., 2018). The genetic distance between seedlings was calculated using ns-SNP data, and followed by Principal Coordinates Analysis (PCoA) in order to plot patterns of genetic divergence in the western hemlock population using GenAlEx (Peakall and Smouse, 2006).

## Results

### Evaluation of *Heterobasidion occidentale* Genes Expressed in Hemlock Stem Tissues Post-inoculation

Phenotypic assessment at 8 months post-inoculation grouped all 60 inoculated seedlings into three categories: death (7%), which died during the experimental stage and no RNA-seq analysis was followed up with these seedlings; diseased (43%), which showed clearly infected symptoms, including abnormal needle-shed, chlorotic foliage, reduction in leader and branch growth; and healthy (50%), which displayed the normal growth as the un-infected (Uif) type similar to the mock-inoculated control (Ctr) seedlings (Supplementary Figure S1). Those diseased seedlings showed infection symptoms at different levels. We classified the infection level with abnormal needle shed below 20% as the quantitative resistant (QR) type, and the infections with abnormal needles shed above 20% up to 90% considered as the susceptible (Sus) type. Dissection of a subset of seedlings with disease symptoms showed discolored wood caused by fungal decay at the inoculation site, while seedlings of both Ctr and Uif types lacked this disease symptom and the wounded inoculation sites were well healed.

**Figure 1:**
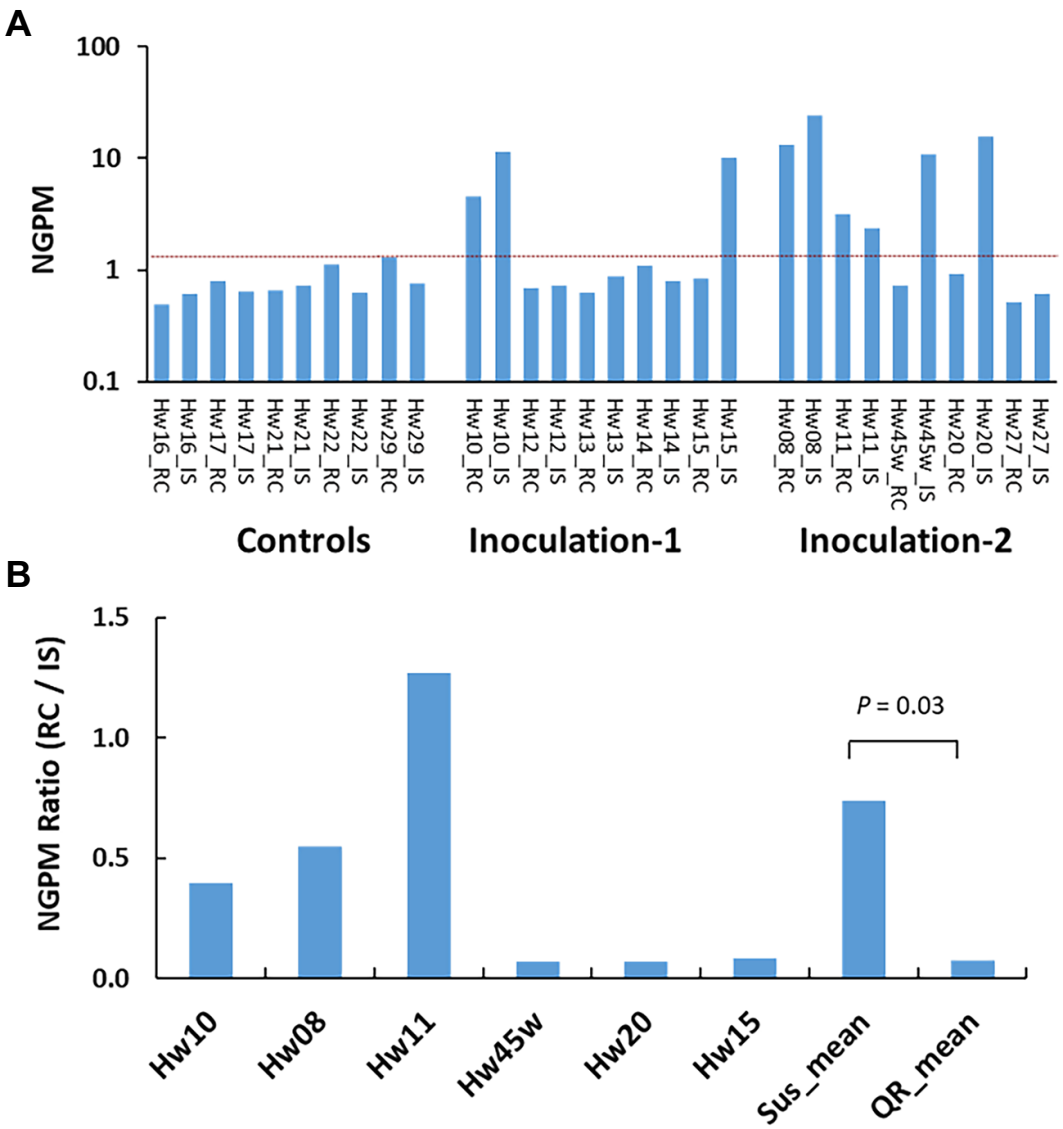
Variations of *Heterobasidion occidentale* genes expressed in western hemlock tissues. Number of expressed genes detected by RNA-seq were normalized as number of genes per million raw reads (NGPM). **(A)** Gene detected in 15 individual seedlings, and each seedling was sampled at the inoculation site (IS) and root collar (RC), respectively. Red dash line separate infected samples from non-infected samples based on the NGPM as measured in the controls (seedlings with mock inoculation). **(B)** Grouping of infected seedlings based on NGPM ratios. Significant difference was detected between susceptible seedlings (Sus) and seedlings with quantitative resistance (QR).

Ten inoculated survivors (three, three, and four seedlings of Sus, QR, and Uif types), and five Ctr seedlings were selected for RNA-seq analysis at about 22 months post-inoculation (Supplementary Table S1). The expressions of *H. occidentale* genes in host tissues were used to estimate disease progress by mapping RNA-seq reads against a *H. occidentale* reference transcriptome. The number of expressed fungal genes in inoculated seedlings at the stem inoculation sites, varied widely from 31 to 1,214. In contrast, only 34–65 fungal genes were detected in the Ctr seedlings with mock inoculation. A similar pattern was observed when fungal transcripts were calculated (Supplementary Figure S2). After normalization for the number of expressed fungal genes per million RNA-seq reads used to map the *H. occidentale* reference transcriptome (NGPM), the Ctr seedlings had NGPM ≤ 1.30 while inoculated seedlings had NGPM values in a range from 0.59 (Hw27) to 36.08 (Hw08). As compared to Ctr seedlings, six inoculated seedlings of Sus and QR types (Hw08, 10, 11, 15, 20, and 45 w) were identified as having been successfully infected with NGPM ≥ 10, while four Uif seedlings (Hw12, 13, 14, and 27) had NGPM ≤ 1.1 (Figure 1A), which is in the same range as the Ctr seedlings did. This confirmed Uif seedlings were not infected, and thus excluded from the gene expression study.

Based on NGPM ratios between the root collar (RC) and the stem inoculation sites (IS), we observed three Sus seedlings (Hw08, 10, and 11) with NGPM ratios (RC/IS) > 0.37. Three QR seedlings (Hw15, 20 and 45w) had NGPM ratios (RC/IS) < 0.09 (Figure 1B), with significant difference between Sus and QR groups (*t*-test, *p* = 0.03).

### *De novo* Assembled Transcriptome

In total over 800 million 100-bp RNA-seq PE reads were generated from 30 cDNA libraries for 15 seedlings, each including the stem tissues sampled at inoculation site and the root collar, separately; and used for *de novo* assembly of the transcriptome (Supplementary Table S1). A preliminary assembly generated 1,193,147 transcripts. The hemlock seedlings for samples collected in the present study were grown in the greenhouse and a large number of trace transcripts likely originated from this environment, particularly from soil that was in contact with the root collars. Removal of trace transcripts by filtering the preliminary assembly at TPM ≥ 1 at least in one sample resulted in 90,730 genes. The longest transcripts were selected for each gene. Based on taxonomic assignment by MEGAN analysis and sequence homology to the databases of other conifers with available genome sequence drafts, a final set of 33 K unique transcripts was generated as a western hemlock reference transcriptome (GenBank Bioproject PRJNA791410), with the average length of 1,428 bp, N50 of 2,438 bp, and total length of 47.9 Mb (Supplementary Table S2). This reference transcriptome was used to detect DEGs between controls and infected seedlings, as well as between Sus and QR genotypes.

### Hemlock DEGs in Response to *Heterobasidion occidentale* Inoculation

**Figure 2:**
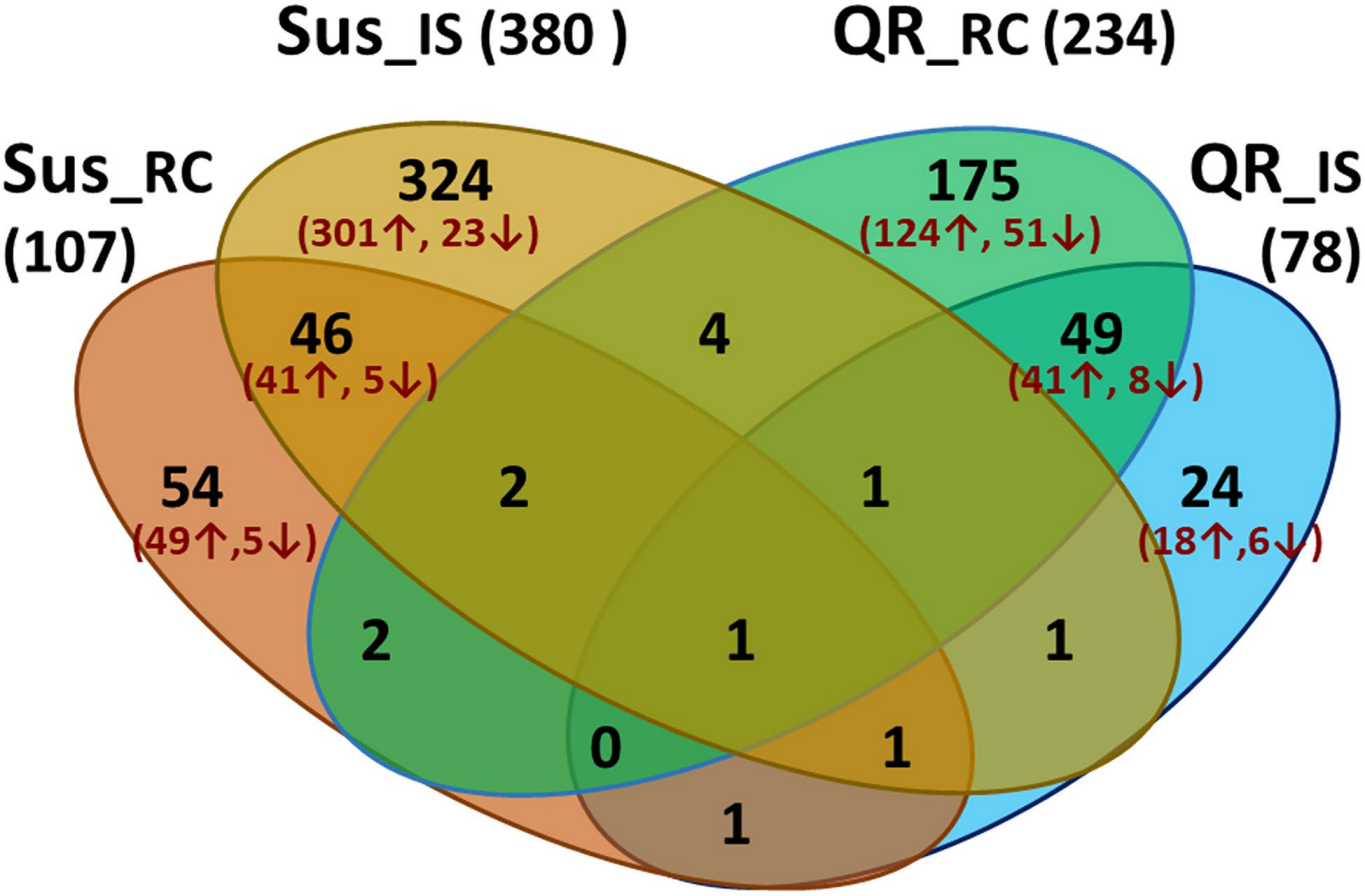
Venn diagram showing numbers of differentially expressed genes (DEGs) during western hemlock–*H. occidentale* interaction. DEGs were identified by transcriptome comparisons between controls (mock-inoculation) and seedlings inoculated by fungus with proportional fold changes ≥ 2 at FDR-corrected *p* < 0.05. Four comparisons are included as susceptible seedlings (Sus), or seedlings with quantitative resistance (QR) versus controls at the inoculation sites (IS) and root collar (RC), respectively.

In order to understand hemlock defense in response to infection by *H. occidentale*, transcriptome profiles of three groups of inoculated seedlings (QR, Sus, and Uif) were compared to controls (Ctr). Host genes were considered as DEGs with a proportional fold change of RPKM ≥ 2 and a cut-off of FDR-corrected *p* < 0.05. As compared to Ctr seedlings with mock inoculation, 78 and 234 were detected in QR seedlings at inoculation sites (IS) and root collar (RC), respectively. Of these DEGs, 183 and 65 DEGs were up- or down-regulated specifically in the QR seedlings (Figure 2).

In Sus seedlings, 380 and 107 DEGs were detected at inoculation sites (IS) or root collar (RC), respectively (Figure 2). Opposite to the QR seedlings, we detected about three time more DEGs at the inoculation site than in the root collar of Sus seedlings. In total, 391 and 33 were up- and down-regulated specifically in the Sus seedlings (Figure 2).

### DEGs Specifically Regulated in the QR Defense Response

**Figure 3:**
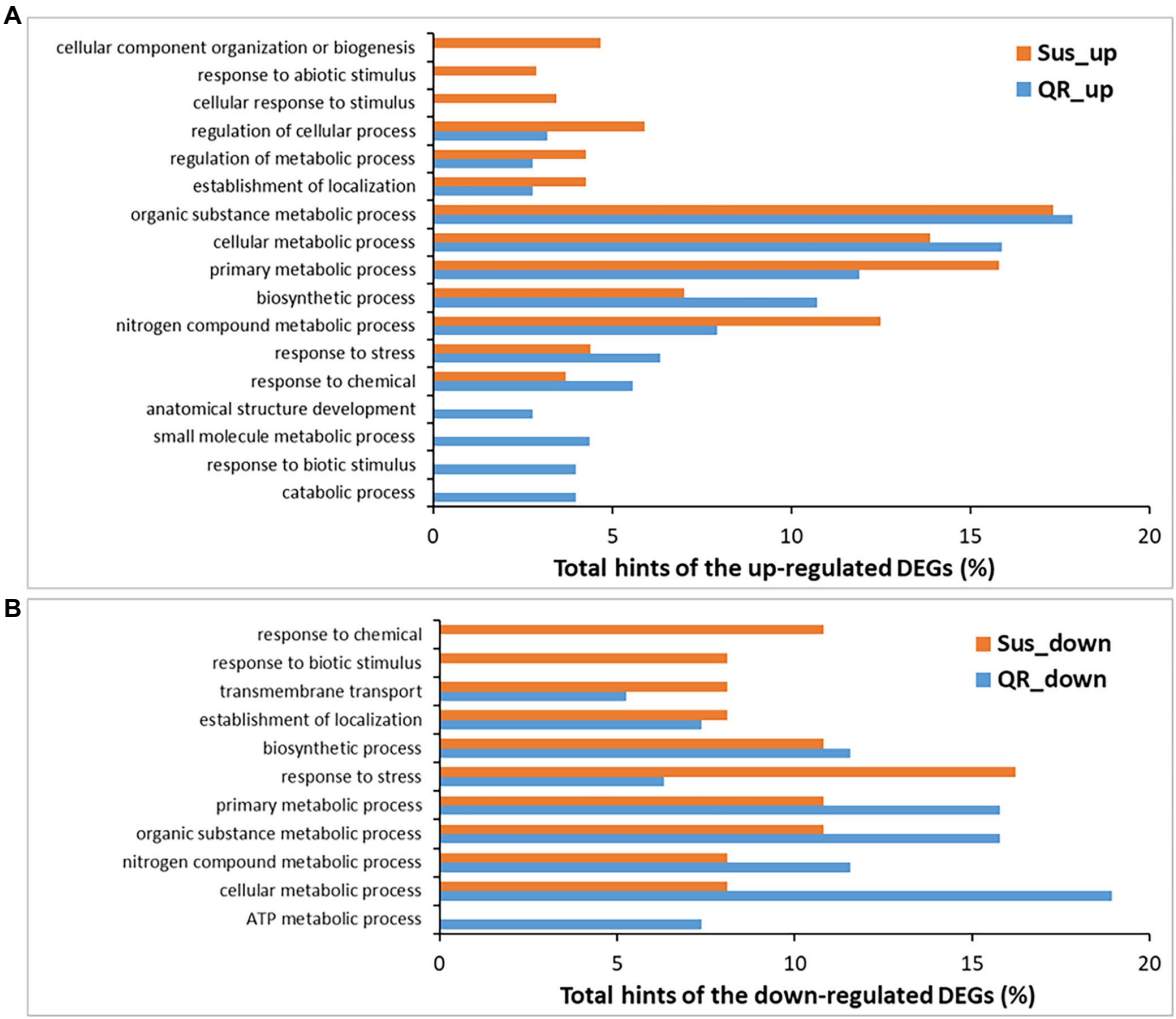
Biological processes (level-3) annotated for differentially expressed genes (DEGs). DEGs were identified in susceptible seedlings (Sus) and seedlings with quantitative resistance (QR) post-inoculation by *H. occidentale*. **(A)** Biological processes for up-regulated DEGs, **(B)** Biological processes for down-regulated DEGs.

To determine genes potentially contributing to host genetic resistance to Annosus root and butt rot disease, DEGs were subjected to gene ontology (GO) analysis. The 183 DEGs up-regulated specifically in the QR seedlings were assigned to 14 GO terms under the category of biological processes (level-3), the most abundant ones including organic substance metabolic process, cellular metabolic process, primary metabolic process, biosynthetic process, nitrogen compound metabolic process, response to stress, and response to chemical. As compared to the Sus seedlings, four GO terms were QR-specific, including catabolic process, response to biotic stimulus, small molecule metabolic process, and anatomical structure development (Figure 3A), suggesting that unique genes responsive to biotic stressors might play important roles in hemlock resistance to infection by the Annosus root and butt rot disease.

Fifty-three QR-up-regulated DEGs showed ≥ 4 fold changes, and encoded putative proteins of the NLR family for plant resistance and components in signaling and metabolisms of plant hormones, including tetratrico peptide repeats (TPRs)-containing proteins, S-adenosyl-L-methionine: salicylic acid carboxyl methyltransferase-like protein (SAMT), and 2-oxoglutarate and Fe_II-dependent oxygenase superfamily protein (2ODO). Others were enzymes participating in the biosynthetic processes such as L-ascorbic acid _AsA (GDP-D-mannose 3′,5′-epimerase), flavonoid (dihydroflavonol reductase, CYP450-like protein), diterpenoid (beta-phellandrene synthase, levopimaradiene/abietadiene synthase), lignin (O-methyltransferase 1), and lignan (phenylcoumaran benzylic ether reductase_PCBER); enzymes in the cellular oxidant detoxification (glutathione S-transferase-like protein GST, glutaredoxin-like protein, phenylcoumaran benzylic ether reductase_PCBER); other downstream defense-responsive proteins (expansin-like proteins, non-specific lipid-transfer protein, dehydrin, early nodulin-like protein, proline-rich arabinogalactan protein; Supplementary Table S3).

Nine biological processes (at level-3) were affected by 65 DEGs specifically down-regulated in QR seedlings. Seven biological process GO terms shared annotation with the up-regulated DEGs, and two other GO terms were also detected (ATP metabolic process and transmembrane transport). The GO term of “ATP metabolic process” was the only one specific to the QR seedlings as compared to Sus seedlings in response to pathogenic infection (Figure 3B). A large proportion of down-regulated DEGs (55 of 65) showed fold changes ≥ 4. In agreement with GO annotation, they were predicted to have putative functions in ATP biosynthetic process (subunits of ATPase), electron transport chain and aerobic respiration (subunits of NADH dehydrogenase, cytochrome oxidases, and cytochrome c oxidases), and photosynthesis (protochlorophyllide reductase). Others included leucine-rich repeat receptor-like protein kinase (LRR-RLK), CYP450s, Hsp70 ATPase ssc1, and several ribosomal proteins (Supplementary Table S4).

### DEGs Specifically Regulated in the Sus Defense Response

There were a set of 391 DEGs up-regulated specifically in the Sus seedlings contributing to 13 biological processes (at level-3). Ten of these were shared with DEGs up-regulated in QR seedlings and three were GO terms specific to Sus seedlings (cellular response to stimulus, response to abiotic stimulus, and cellular component organization or biogenesis; Figure 3). Fifty-four DEGs were up-regulated with fold changes ≥ 4, and included two LRR-RLKs and three NLRs with potential roles in initial perception of pathogen invasion. There were three signaling factors (glutamate receptor, ARF-GAP domain protein, and metacaspase), two transcriptional factors (TF) of the basic helix–loop–helix (bHLH) DNA-binding superfamily and the LOB domain-containing family. Additionally, there were five proteins in the minichromosome maintenance complex (MCM) for genomic DNA replication, four enzymes for polysaccharide catabolic process (glycosyl hydrolase family 9 protein, glucan 1,3-beta-glucosidase A, glycosyl hydrolase, and mannan O-acetyltransferase), three transmembrane transporters (major facilitator superfamily protein, purine permease, sulfate/thiosulfate import ATP-binding protein, heavy metal transport/detoxification superfamily protein), and three expansins for plant-type cell wall loosening. Other defense-responsive components included peroxidases, amine oxidase, DUF1218-containing protein, enoyl-CoA hydratase/isomerase family protein, etc. (Supplementary Table S5).

There were a set of 33 DEGs down-regulated specifically in the Sus seedlings and involved in 10 biological processes (at level-3), eight of these were shared with DEGs down-regulated in QR seedlings and two GO terms (response to biotic stimulus, and response to chemical) were Sus-specific (Figure 3), suggesting that the down-regulation of responses to both biotic and chemical stimuli is one molecular mechanism contributing to western hemlock’s susceptibility to Annosus root and butt rot disease. Of the Sus specifically regulated DEGs, 24 showed fold changes ≥4, with a range of putative functions, such as amino acid permease, embryo-abundant protein, polygalacturonase, peroxidase, peptidase, and others (Supplementary Table S6).

### Gene Ontology Term Enrichment Analysis of DEGs

**Figure 4:**
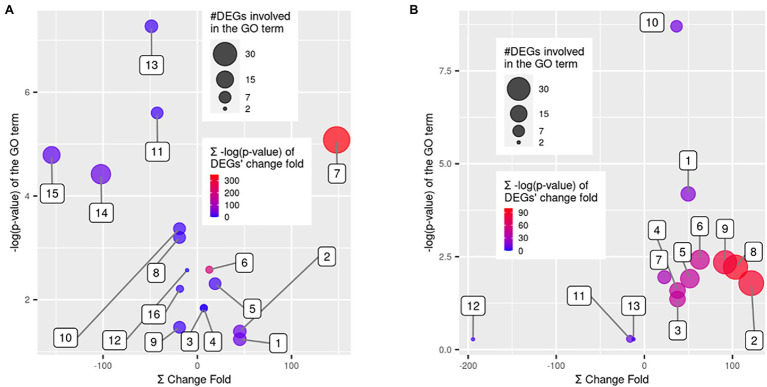
GO term enrichment analysis for the differentially expressed genes (DEGs). Scatter plots show the False Discovery Rate (FDR)-corrected *p* values of GO term enrichment analysis using Fisher’s exact test vs. a sum of fold changes of the DEGs. The enriched GO terms were over-represented in DEGs as compared to the whole western hemlock reference transcriptome. Bubble sizes indicate the number of DEGs involved in each GO term, and bubble colors indicate a sum of the FDR-corrected p values for the statistical significance of DEGs’ fold changes. **(A)** GO terms enriched in resistant seedlings, including (1) terpenoid metabolic process, (2) terpenoid biosynthetic process, (3) cinnamic acid biosynthetic process, (4) cinnamic acid metabolic process, (5) benzene-containing compound metabolic process, (6) GDP-mannose 3,5-epimerase activity, (7) oxidoreductase activity, (8) steroid metabolic process, (9) response to wounding, (10) xylem and phloem pattern formation, (11) cellular respiration, (12) iron coordination entity transport, (13) electron transfer activity, (14) oxidoreductase activity, (15) transmembrane transporter activity, and (16) proton-transporting ATP synthase complex. **(B)** GO terms enriched in susceptible seedlings, including (1) DNA replication, (2) cellular component organization, (3) external encapsulating structure organization, (4) cell wall organization, (5) DNA-binding transcription factor activity, (6) hydrolase activity, hydrolyzing O-glycosyl compounds, (7) pectinesterase activity, (8) cell periphery, (9) plasma membrane, (10) MCM complex, (11) plant-type cell wall modification, (12) basic amino acid transport, and (13) killing of cells of other organism.

Each group of DEGs with up- or down-regulation in QR and Sus seedlings as compared with control seedlings was subjected to GO enrichment analysis using the whole transcriptome as reference. For the 183 upregulated genes in the QR seedlings, seven GO terms were significantly enriched (FDR-corrected *p* < 0.05), including five biological process (terpenoid biosynthetic process, terpenoid metabolic process, benzene-containing compound metabolic process, cinnamic acid biosynthetic process, and cinnamic acid metabolic process) and two molecular functions (GDP-mannose 3,5-epimerase activity and oxidoreductase activity; Figure 4). For the 65 down-regulated genes in the QR seedlings, nine GO terms were significantly enriched (FDR-corrected *p* < 0.05), including five biological processes (cellular respiration, xylem and phloem pattern formation, steroid metabolic process, response to wounding, and iron coordination entity transport), three molecular functions (electron transfer activity, oxidoreductase activity, and transmembrane transporter activity), and one cellular component (proton-transporting ATP synthase complex; Figure 4A).

For the 391 upregulated genes in the Sus seedlings, the most significantly enriched GO terms included four biological process categories (cellular component organization, DNA replication, external encapsulating structure organization, and cell wall organization), three molecular functions (DNA-binding transcription factor activity, hydrolase activity-hydrolyzing O-glycosyl compounds, and pectinesterase activity) and three cellular components (cell periphery, plasma membrane, and the minichromosome maintenance (MCM) protein complex; Figure 4B). Although no significant enrichment (FDR-corrected *p* < 0.05) was observed for the 33 down-regulated DEGs in the Sus seedlings, some GO terms (*p* < 0.001 before FDR adjustment) were worthwhile to mention, such as basic amino acid transport, killing of cells of other organism, and plant-type cell wall modification, with the implication that these biological processes are manipulated by the pathogen in order to cause host susceptibility to disease (Figure 4B).

### Confirmation of RNA-Seq Data for DEGs by RT-qPCR

To further confirm transcriptomes profiled by RNA-seq data, RT-qPCR was performed to evaluate gene expression patterns for 18 selected genes (Supplementary Table S7). Fold changes of gene expression were calculated between Ctr seedlings and Sus or QR seedlings, and fold change correlations were evaluated to estimate concordance in gene expression intensities between RNA-seq and RT-qPCR. A significant fold change correlation was observed between RT-qPCR and RNA-seq data for all 18 analyzed genes (Pearson correlation *R* = 0.4629, *p* = 4.2 × 10^−5^, Supplementary Figure S3A). To quantify potential discrepancies between RNA-seq and RT-qPCR, Pearson correlations between RPKM values of RNA-seq and relative quantification (RQ) of RT-qPCR were examined across all samples for each gene. This analysis revealed 11 as concordant genes and 7 as non-concordant genes with agreement, or disagreement of expression patterns between RNA-seq and RT-qPCR, respectively (Supplementary Figure S3B). The concordant genes included the DEGs identified above, such as those encoding plant cysteine oxidase 3 (PCO3), GMC oxidoreductase, and CYP450-like, trichome birefringence-like 25 (TBL25) protein (Supplementary Figure S4).

### Genetic Diversity of Hemlock Trees

**Figure 5:**
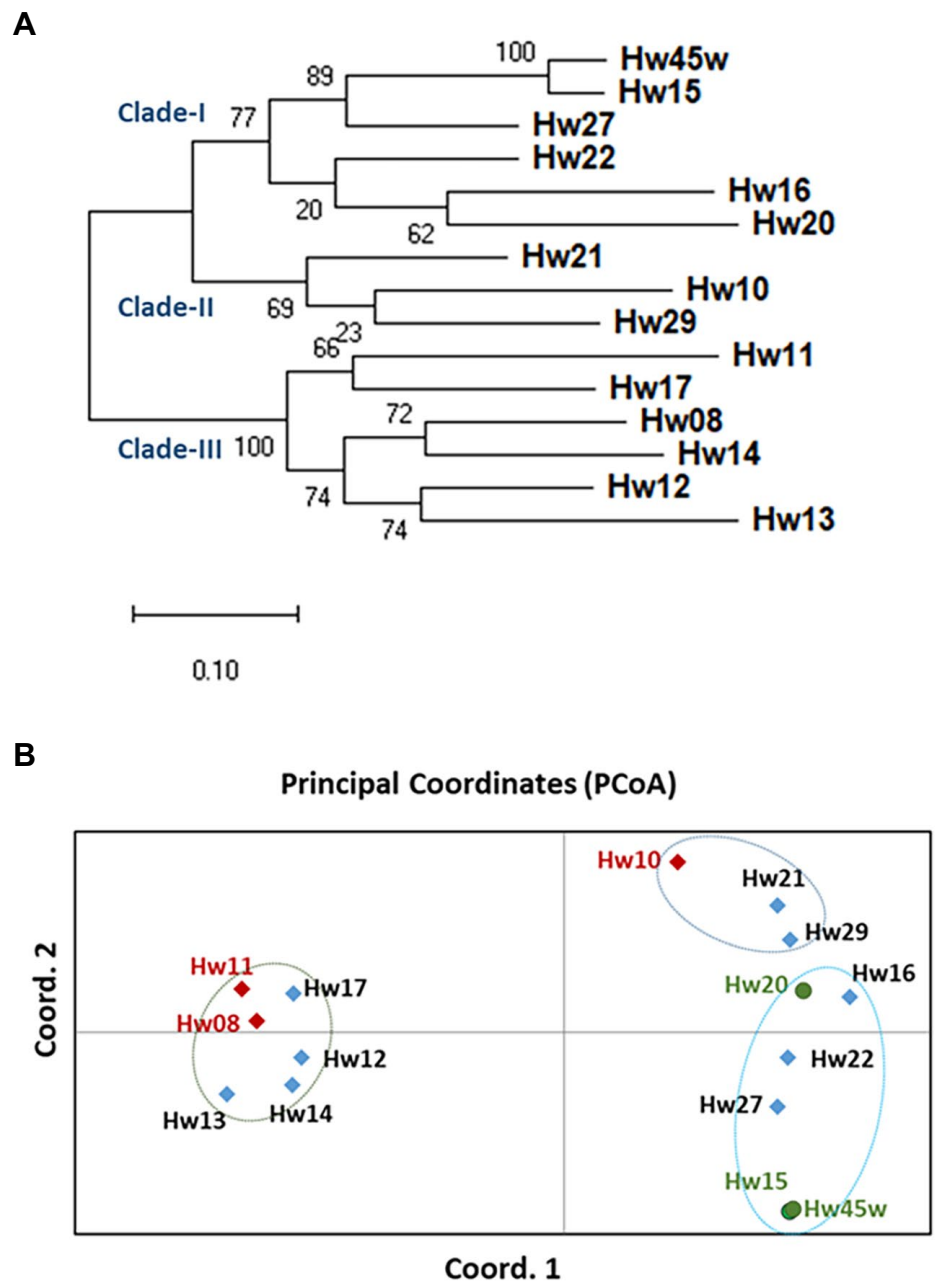
Genetic divergence and evolutionary relationships among western hemlock individuals. **(A)** The phylogenetic tree constructed using maximum likelihood (ML) method. A total of 1,043 ns-SNPs extracted within 599 defense- and resistance-related genes were included for alignment of polymorphic loci. The bootstrap values determined with 100 repeats are represented close the branching nodes. **(B)** Principal Coordinates Analysis (PCoA) of western hemlock samples based on ns-SNPs data (variance of axis 1: 39.95%; variance of axis 2: 12.92%).

A total of 1,043 ns-SNPs were detected in 599 genes with annotation as related to disease resistance and plant defense responses in the hemlock population (Supplementary Tables S8 and S9). Based on alignment of these SNP loci, phylogenetic tree constructed using maximum likelihood (ML) analysis clearly grouped the seedlings into two main groups and three clades with good bootstrap supports ranging from 64 to 100%, indicating a high degree of close genetic relationship within each clade. The QR seedlings (Hw15, 20, and 45w,) were grouped into Clade-I while the Sus seedlings (Hw08, 10, and 11) were each relatively independent, and distributed in clade-II and clade-III, respectively. One Uif (Hw27) and two Ctr (Hw16 and 22) seedlings were grouped with QR seedlings in Cluster-I, and other three Uif (Hw12, 13, and 14) and three Ctr (Hw17, 21 and 29) seedlings were grouped with Sus seedlings in either Cluster-II or clade-III (Figure 5A).

Based on a Nei’s unbiased genetic distances among samples, a two-dimensional PCoA plot revealed the evolutionary relationships between seedlings (Figure 5B), explaining 40 and 13% of the total variation in the first and second principal coordinates, respectively. Consistent with the genetic divergence pattern as detected by the phylogenetic analysis, PCoA also divided individual samples into two main groups. Interestingly, the QR seedlings were placed close to each other along the first principal coordinate, while three Sus samples were well separated from each other along both the first and second principal coordinates. The result of both phylogenetic analysis and PCoA suggests that resistant and susceptible genotypes have different evolutionary origins and adaptive paths.

## Discussion

Root diseases of forest trees are considered to be the most destructive tree diseases in forest ecosystems. Annosus root and butt rot caused by *Heterobasidion* spp. complex has affected many important forest tree species, including a wide range of conifers (such as hemlocks, firs, pines, and spruces), as well as several broadleaf tree species (Asiegbu et al., 2005). The BC hemlock breeding program has carefully collected seeds from a set of specific elite western hemlock parental trees to develop composite seed families for genetic gains with higher forest performance (Cappa et al., 2015; Cartwright and King, 2021). Although information on germplasm origin is useful for genetic diversity assessment, genetic diversity is largely unquantified in western hemlock. In particular, genetic information on host resistance types and level of resistance against invasion by pests or pathogens is not available in western hemlock populations. This study evaluated disease progression and host transcriptomic reprogramming following fungal inoculation, as well as genetic diversity and DNA variations in plant defense- and resistance-related genes in a composite seed family.

Based on phenotypic assessment of disease symptom-related traits and expression profiles of *H. occidentale* genes, variability in disease development was detected from disease susceptibility to quantitative resistance at different levels among trees post-inoculation, dividing the inoculated seedlings into three groups. Those inoculated but not infected (Uif) seedlings showed healthy growth, like the mock-inoculated (Ctr) seedlings. It is noticed that a small set of *H. occidentale* genes were detected at low levels in both Uif and Ctr seedlings, probably originated from environmental microbial contamination or *in silico* random occurrences (Davis et al., 2018). Contaminating microbial reads are commonly detected in the host reads sequenced by RNA-seq or other metagenomic sequencing approaches (Park et al., 2019). Pre-sequencing contaminants are unavoidable in environmental samples. Further computational approaches may help determine if the contaminant reads in the western hemlock metatranscriptomic datasets were originated from *H. occidentale*, or other microorganisms (such as endophytes and soil microbiome).

The wounding-based *Heterobasidion* inoculation method was widely used, but usually showed no infection in a considerable proportion of inoculated seedlings (Liu et al., 2022). These inoculated but not infected seedlings were considered as failed infections (Arnerup et al., 2010), or as a resistant trait with full fungal exclusion (Lind et al., 2014). Whether complete immunity to *H. occidental* is present in western hemlock populations requires the development of a more effective inoculation protocol. In western hemlock, contrasting genotypes of the QR and Sus seedlings were further supported by RNA-seq-based transcriptome profiling and phylogenetic analysis. Genetic variation in defense- and resistance-related genes provided a direct link between genotypes and phenotypes for the disease resistance and susceptibility. Similarly, symptomatic trees were found with higher abundance of *Heterobasidion*-specific transcripts than the asymptomatic trees, leading to the identification of Norway spruce candidate genes as markers for higher resistance against Annosus root and butt rot disease (Kovalchuk et al., 2019).

Our RNA-seq-based transcriptome profiling clearly demonstrated that western hemlock’s defense against *H. occidentale* was activated through reprogramming of its transcriptome, showing significant differentials between QR and Sus seedlings. A number of DEGs were identified during the compatible interaction of western hemlock with *H. occidentale* by RNA-seq analysis. RT-qPCR analysis verified that the majority of the selected genes displayed similar transcript expression patterns as detected by RNA-seq. At the transcriptome level, of the total expressed genes 80–85% are defined as concordant genes, showing consistent expression patterns between RT-qPCR and RNA-seq data. The others are termed as non-concordant genes with conflicting expression patterns between RT-qPCR and RNA-seq, having lower expression levels, smaller size, and fewer exons as compared to the concordant genes (Everaert et al., 2017).

### Molecular Mechanisms and Candidate Genes Underlying Western Hemlock QR Against *Heterobasidion occidentale*

We detected a set of host DEGs with transcripts significantly regulated during the interactions between the QR seedlings and the fungus. Annotation of the DEGs specifically regulated in the QR seedlings found a series of biological processes involved in resistance against Annosus root and butt rot disease. Western hemlock genes of the NLR and LRR-RLK families were differentially expressed with some members regulated in QR seedlings and others in Sus seedlings. NLRs are well-known receptors of the plant immune system, which recognize microbial effectors, playing divergent roles in both pattern-triggered immunity (PTI) and effector-triggered immunity (ETI) that enable plants to activate downstream molecular defense responses for trait developments of major gene resistance or QR (Adachi et al., 2019; Zhai et al., 2022). RLKs (including LRR-RLKs) play a central role in sensing various signals to regulate plant development and defense responses to abiotic and biotic stresses, including attack by pests and pathogens (Chen, 2021).

Perception of the pathogenic signals by plant immune system triggers a network of signaling pathways to activate downstream defenses responses. Several QR-upregulated genes were annotated as signaling components, including SAMT, 2ODO, and TPR-containing proteins, involved in plant hormone-mediated signaling (Schapire et al., 2006; Caarls et al., 2017). SAMT catalyzes salicylic acid (SA) to produce Methyl salicylate (MeSA), a volatile compound for a long-distance mobile signal in stress defense and systemic acquired resistance (SAR) mechanism (Shulaev et al., 1997; Park et al., 2007). SMAT genes were the targets for genetic manipulations to biotechnologically improve disease resistance (Zou et al., 2021). SNP markers of the MeSA biosynthetic genes associated with low and high MeSA content were explored for application in molecular breeding of birch species (Singewar et al., 2020). Different members of the 2ODO superfamily are involved in oxygenation/hydroxylation of multiple plant hormones including gibberellic acid, auxin, salicylic acid, and jasmonic acid (Caarls et al., 2017). Two TPR-containing genes (*GmSNAP11* and *GmSNAP18*) contributed to soybean QR to nematodes (Cook et al., 2012; Lakhssassi et al., 2017).

In addition, we observed downstream regulation of a wide set of host genes as part of the defense response in QR seedlings. The downstream responsive genes were annotated as enzymes for enhanced biosynthetic processes of various secondary metabolites (such as L-ascorbic acid, cinnamic acid, flavonoids, terpenoids, lignins, and lignans). As one of the most abundant antioxidants in plants, ascorbic acid protects cells from the damage caused by reactive oxygen species (ROS), playing an important role in the induced resistance (IR) to pathogens (Boubakri, 2017). Plant GDP-D-mannose epimerase (GME) converts GDP-D-mannose to GDP-L-galactose, a precursor of both L-ascorbate and cell wall polysaccharides; and its over-expression enhances tolerance to abiotic stresses by increasing ascorbate accumulation (Ma et al., 2014). Cinnamic acid, on the other hand, is a toxic phenolic acid which makes plants less resistant to pathogens and causes oxidative damage when its concentration increases (Huang et al., 2013; Guo et al., 2020).

Among other secondary metabolites, flavonoids comprise a large group of plant polyphenolic compounds and are well known for their multiple functions in plant growth and defense response against pathogens, herbivores, and other environmental stresses (Panche et al., 2016). The Norway spruce (*Picea abies*) QR to *H. annosum s.l.* was associated with an allele that encodes an enzyme of the flavonoid biosynthetic pathway (Nemesio-Gorriz et al., 2016). The lignosuberzed boundary zone was formed in the bark adjacent to the *Heterobasidion* inoculation site, playing an important role in the induced defense response in Norway spruce (Elfstrand et al., 2020). Terpenoids are well-characterized defense compounds in conifers (Bohlmann, 2012; Novick et al., 2012), with potential as biomarkers for the evaluation of Scott pine tolerance against *H. annosum* (Mukrimin et al., 2019). As an aromatic polymers in the cell walls, the lignin-deposits function as a physical barrier to trap pathogens and terminate their growth, leading to disease resistance in plants (Lee et al., 2019). Lignan is a group of phenylpropanoids and PCBER is involved in lignan synthesis by reducing phenylpropanoid dimers to form antioxidants that protect plants against oxidative damage (Niculaes et al., 2014). In alignment with these previous studies, the enhanced synthesis of these secondary metabolites can provide antifungal components that limit *H. occidentale* growth and proliferation inside the stem tissues of the western hemlock QR seedlings. Other defense-responsive components include non-specific lipid-transfer proteins which are well known for their role in plant QR against various pathogens (Liu et al., 2015). Functional verification of these identified DEGs in future studies, will allow for the desired alleles of these QR-related genes to be selected for phenotypic performance to improve disease resistance in western hemlock breeding.

### Candidate Genes Contributing to Hemlock Susceptibility to *Heterobasidion occidentale*

The transcriptome reprogramming of the Sus seedlings considerably contrasted with that of the QR seedlings post-inoculation by *H. occidentale*. In the Sus seedlings, the expression of defense-responsive genes was regulated mainly at the infection site, while in the QR seedlings, it appeared mainly at the root collar, a distance away from the infection site. The Sus-specific DEGs were annotated with functions mainly in response to abiotic stimuli, while the QR-specific DEGs were annotated with functions mainly in response to biotic stimuli. The Sus seedlings had significantly enriched GO terms in biological processes of cell wall organization, DNA replication, and cellular component organization, while the QR-seedlings showed significantly enriched GO terms involved in the metabolic and biosynthetic processes of terpenoids and cinnamic acid. The genes for “killing of cells of other organism, and plant-type cell wall modification” were consistently highly suppressed in the Sus seedling, suggesting that western hemlock susceptibility (S) gene might be hijacked by fungal pathogens, facilitating infection and supporting compatible tree-pathogen interaction (Zhang et al., 2021).

Understanding molecular interactions of host S genes with pathogenicity–related factors is an important strategy for developing durable resistance in plant breeding (Gorash et al., 2021). However, neither R nor S gene has been functionally characterized in a forest species yet, including western hemlock. As the first step to identify western hemlock S genes that confer successful fungal infection, this study profiled transcriptomes of Sus seedlings post-*H. occidentale* inoculation, and a large set of DEGs were identified with annotated functions. Among them, unique NLR and RLK homologs were detected with upregulation by *H. occidentale* in either QR or Sus seedlings. In addition to their roles in disease resistance, the plant LRR-RLKs and NLRs families also contain members functioning as S genes. As plant pattern recognition receptors (PRRs), they perceive pathogen/microbe-associated molecular patterns (PAMPs/MAMPs), including pathogenic effectors and other pathogenicity-related factors. Successful pathogens attenuate plant PTI by delivering various effectors, resulting in effector-triggered susceptibility for completion of disease development in susceptible plants (Alhoraibi et al., 2019). Comparison of S genes characterized in other plants (Van Schie and Takken, 2014), with up-regulated DEGs in the Sus seedlings revealed additional S candidates, such as protein kinases of various families, ARF-GAP domain proteins, bHLH transcriptional factors, metacaspase, expansin, CYP450-like, and phospholipase in western hemlock. For instance, due to its immunomodulatory function, metacaspase was suggested as a useful target for marker-assisted breeding or improvement of disease resistance by CRISPR-Cas mutagenesis (Hander et al., 2019).

In addition to RNA-seq, genome-wide association analysis (GWAS) was also used to identify the SNPs and the candidate genes significantly associated with Norway spruce susceptibility to *H. parviporum* (Mukrimin et al., 2018). A previous study characterized a large set of secreted proteins as candidate effectors in the *H. occidentale* transcriptome (Liu et al., 2018). Effectors from pests/pathogens have capacity to suppress the NLR-dependent host resistance by directly binding to the NLRs or other molecular mechanisms (Derevnina et al., 2021). Interaction of host S genes with pathogens’ effectors activates effector-triggered susceptibility (ETS; Gouveia et al., 2016). Effectors have emerged as a powerful tool to identify plant PRRs and to search for novel R genes for genomics-based breeding (Domazakis et al., 2017). Meanwhile, identification of putative S genes by searching the available genomic databases provide targets for further reverse genetics studies in non-model species. The western hemlock transcriptome *de novo* assembled in this study provides a useful resource to document conifer orthologues of well-characterized S genes. Future studies involving functional analysis can aim to determine if the candidate western hemlock S genes and *H. occidentale* effectors interact with each other.

### Integration of Genomic Resources for Hemlock Resistance Breeding

Assessment of population genetic diversity is necessary for making decisions on utilization and preservation of species resources, and now it can be done with the aid of genomic approaches in a fast, effective, and accurate way. We have successfully mined thousands of SNPs within defense- and resistance-related genes from RNA-seq data using a pipeline from multiple bioinformatics tools. Although paralogy or splicing did cause artificial SNPs in the RNA-seq-based datasets, the potential and accuracy of RNA-seq as an efficient way to genotype SNPs were well verified by other genomic approaches in different plants (Rogier et al., 2018). Using the ns-SNPs detected with defense- and resistance-related genes, this study evaluated the genetic diversity of a composite seed family, indicating that selection of parental trees in previous breeding programs covered a high level of genetic diversity from wild western hemlock stands. An ML-based phylogenetic tree revealed a clear separation between individuals, grouping them into three distinct evolutionary clades with long genetic distances. This high level of genetic diversity in western hemlock populations is in agreement with their breeding history (Cartwright C, personal communication). The observed genetic structure can be explained mainly by the geographic distribution of the parental trees since the origins of the composite seed family had wide geographic range in BC and WA. Accurate estimation of the genetic diversity and genetic structure across a large population will provide a foundation for future western hemlock breeding. Although geographic origin provides useful information for the initial assessment of germplasm genetic diversity, omics technologies can distinguish these germplasms at fine resolution across the genome. This is important especially for genetic dissection of plant QR to pathogens. Large genetic diversity with continuous resistance level in plant populations is a key prerequisite for successful selection of future plant generations for durable disease resistance in breeding programs (Sniezko and Liu, 2022).

Phylogenetic analysis and PCoA analysis revealed that QR seedlings were in close proximity within one clade, and evolutionarily distinct from Sus seedlings that were widely distributed in other two phylogenetic clades. Phylogenetic analysis and PcoA results also suggest that one Uif and two Ctr seedlings may be resistant because they were closely grouped with QR seedlings in the clade-I. However, it needs to be confirmed by a future inoculation trial with sufficient clonal replicates. The current genetic analyses results suggest that a fraction of the composite seed family can be selected in a new breeding cycle to gain higher levels of genetic resistance to Annosus root and butt rot disease. Conventional selection of disease-resistant trees is done by inoculation of progeny, followed by phenotypic assessment, taking many years to complete with high costs for forest tree breeders (Sniezko and Liu, 2022). Advent of novel genomics resources and breeding tools is recognized as a revolutionary force that has changed conventional breeding practices in the last two decades. Although the integration of genomic resources into breeding practices is still challenging, some case studies demonstrated that marker-assisted selection (MAS) was able to predict disease resistance in wild stands and in their progeny by genotyping without phenotyping (Liu et al., 2020).

Furthermore, our analysis of genotypic differences between QR and Sus seedlings was based on ns-SNPs of defense- and resistance-related genes that cause changes to amino acids with putative functional variation. The identified genes and functional alleles allow future development of MAS tools for application in breeding of western hemlock resistance against *H. occidentale*. R genes of the plant NLR and RLK families have been used to develop MAS tools in a wide range of crop species, including conifer trees (Liu et al., 2021). A large set of SNP loci was detected within western hemlock defense- and resistance-related genes, providing genomic regions to target for their verification as MAS tools for future hemlock breeding. QR is usually polygenic. Genotyping of a few markers, or an array of DNA markers, allows selection of quantitative trait loci (QTLs) underlying QR-related phenotypic traits in plant breeding, even for those QTLs with small phenotypic effects (Das et al., 2017). Resistant genotypes can be selected as new parents for the next generation with better desired traits through application of such genomics-based tools and technologies. Identification of the resistance genotypes could reduce management cost and improve collection, selection, and maintenance of germplasm resources. Meanwhile, it may be necessary for western hemlock breeding to incorporate additional genetic components by utilizing germplasms with full coverage of the species’ geographical distribution. Western hemlock occasionally hybridizes with mountain hemlock (*T. mertensiana* Bong. Carr.), generating a hybrid (*Tsuga x jeffreyi* Henry) in natural populations (Little, 1979). As *H. occidentale* is native to the western regions of North America, potential interspecific hybridization between close-related hemlock species may introduce novel resistance to this fungal pathogen.

During the long history of the arms races between western hemlock and *H. occidentale*, host resistance and susceptibility to Annosus root and butt rot disease have probably been kept in equilibrium until the emergence of disease outbreaks with new virulent pathogenic races which become dominant across the landscape. However, local adapted populations are currently challenged by changing climate conditions (Sturrock et al., 2011), and emergence of virulent and more aggressive pathogenic races which are expanding their range in consequence of the new environment (Seidl et al., 2017). Nevertheless, genomic knowledge on tree adaptive diversity and flexibility is making selection of elite genotypes more efficient for improved resistance by genome-based predictions of the adaptive traits. Although the functions of the resistance candidate genes await to be confirmed in western hemlock, their defense-responsive expression post-infection by *H. occidentale,* and association with resistance phenotypes demonstrate that they are attractive targets for comprehensive characterization of western hemlock resistance to Annosus root and butt rot disease. Although sample size was relatively limited in this study, the identification of a set of the SNPs within defense- and resistance-related genes suggests useful targets for fine dissection of the genomic architecture to reveal the complex interactions of genotype–phenotype-environment when larger populations are available for GWAS and QTL mapping.

In conclusion, this study examined the phenotypic and genotypic spectrum of western hemlock susceptibility and resistance to Annosus root and butt rot disease. The molecular defense mechanisms and fundamental genetic variation uncovered in this research can be integral to future western hemlock breeding programs. Understanding transcriptome reprogramming in response to pathogenic invasions and identification of the candidate genes for disease resistance are clearly pertinent to improving forest health and productivity. The allelic variants identified within resistance-related genes highlight genomic resources for developing MAS tools by targeting a set of candidate genic ns-SNPs. Thus, these current findings provide beneficial insight into tree-pathogen interactions and valuable information for decision-making in breeding programs and silvicultural operations. Future evaluation of allelic contributions of the candidate genes to the development of resistance traits is fundamental in designing new genomics-based breeding strategies.

## Author’s Note

Annosus root and butt rot caused by *Heterobasidion* spp. complex has affected many important forest tree species, including a wide range of forest conifers and broadleaf species. The BC breeding program has selected parent trees from wild stands of western hemlock (*Tsuga heterophylla*) to develop seed orchards for genetic gain with higher forest performance. However, in western hemlock populations, genetic information on host resistance types and levels against the Annosus root and butt rot disease is unknown. In this study, we evaluated the disease progression and the reprogramming of the host’s transcriptome following inoculation with *Heterobasidion occidentale* and detected seedlings with quantitative resistance. The resistant seedlings showed a unique molecular defense response as compared with that of susceptible seedlings in a western hemlock composite seed family. The susceptible and resistant seedlings were well separated based on their transcriptomic defense profiles and phylogenetic analysis. Furthermore, we detected genetic variation in defense- and resistance-related genes, providing a direct link between genotypes and phenotypic disease resistance and susceptibility. Therefore, resistant genotypes can be selected in a new breeding cycle to gain higher levels of genetic resistance to Annosus root and butt rot disease. The allelic variants identified within resistance-related genes highlight genomic resources to develop molecular tools for marker-assisted selection for future breeding applications. Our findings provide beneficial insight on western hemlock–*H. occidentale* interactions and useful information for decision-making in breeding and silvicultural operations.

## Data Availability Statement

The datasets presented in this study can be found in online repositories. The names of the repository/repositories and accession number(s) can be found at: https://www.ncbi.nlm.nih.gov/bioproject/?term=PRJNA791410.

## Author Contributions

J-JL designed the research, contributed to data analysis, and wrote the manuscript. AZ performed RT-qPCR and data analysis. YX contributed SNP data and genetic diversity analysis. SS contributed fungal isolate and seedling inoculation. CC contributed plant materials and input on plant breeding. BR performed transcriptome assembly and RNA-seq data analysis. All authors contributed to the article and approved the submitted version.

## Funding

This study was funded in part by the CFS Pest Risk Management Project.

## Conflict of Interest

The authors declare that the research was conducted in the absence of any commercial or financial relationships that could be construed as a potential conflict of interest.

## Publisher’s Note

All claims expressed in this article are solely those of the authors and do not necessarily represent those of their affiliated organizations, or those of the publisher, the editors and the reviewers. Any product that may be evaluated in this article, or claim that may be made by its manufacturer, is not guaranteed or endorsed by the publisher.

## Footnotes

^1^ http://www.usadellab.org/cms/?page=trimmomatic

^2^ https://www.biobam.com/omicsbox

^3^ http://revigo.irb.hr/

## Supplementary Material

Supplementary Table S1 A list of seedlings, stem tissues, and cDNA libraries used for RNA-seq analysis.

Supplementary Table S2 Statistics of de-novo assembly of RNA-seq reads to generate a western hemlock reference transcriptome.

Supplementary Table S3 Differentially expressed genes (DEGs) upregulated in seedlings with quantitative resistance (QR) post infection by Heterobasidion occidentale.

Supplementary Table S4 Differentially expressed genes (DEGs) downregulated in seedlings with quantitative resistance (QR) post infection by Heterobasidion occidentale.

Supplementary Table S5 Differentially expressed genes (DEGs) upregulated in susceptible (Sus) seedlings post infection by Heterobasidion occidentale.

Supplementary Table S6 Differentially expressed genes (DEGs) downregulated in susceptible (Sus) seedlings post infection by Heterobasidion occidentale.

Supplementary Table S7 Primers used for quantitative reverse transcription PCR (RT-qPCR).

Supplementary Table S8 A list of genes used for genetic diversity study.

Supplementary Table S9 A list of non-synonymous single nucleotide polymorphisms (ns-SNPs) used for genetic diversity analysis.

Supplementary Figure S1 Assessment of disease symptoms grouped all seedlings inoculated using *Heterobasidion occidentale* into four types. **(A)** Healthy seedlings showed normal growth similar as control seedlings, considered as inoculated but not infected (Uif) type. **(B)** Infected seedlings showed < 20% of abnormal needles, considered as quantitative resistant (QR) type. **(C)** Infected seedlings showed 20% ~ 90% of abnormal needles, considered as susceptible (Sus) type. **(D)** Seedlings died before assessment, and they were not subjected to RNA-seq analysis.

Supplementary Figure S2 Numbers of *H. occidentale* genes and transcripts detected in western hemlock tissues by RNA-seq analysis.

Supplementary Figure S3 Correlation of gene expression levels between measurements by RNA-seq and RT-qPCR. **(A)** Scatterplot illustrating correlation of fold changes measured between controls (mock inoculation) and susceptible seedlings (Sus), or seedlings with quantitative resistance (QR). Relative transcript expression levels were measured by RNA-Seq-based RPKM and RT-qPCR analyses for 18 selected genes. Fold change of transcript levels were made by comparison of infected seedlings with control seedlings with mock-inoculation. The correlation trend line (red dash) shows a significant positive correlation across all 18 tested genes (Pearson *R* = 0.46, *p* = 4.2 × 10^−5^). **(B)** Correlation of relative quantity (RQ) as measured by RT-qPCR and RPKM as measured by RNA-seq in each tested sample. Transcript expression levels were compared for each of 18 genes. Eleven showed significant positive correlation and classified as concordant genes, while the other seven showed non-significant or negative correlations and classified as non-concordant genes. One to three stars indicate significance of correlations at *p* < 0.001, 0.01, and 0.05, respectively.

Supplementary Figure S4 Correlation of fold changes for four differentially expressed genes (DEGs) as measured between controls (mock inoculation) and susceptible seedlings (Sus), or seedlings with quantitative resistance (QR). They were annotated as PCY450-like, trichome birefringence-like, GMC oxidoreductase, and plant cysteine oxidase, respectively.
