# Supplementary figures and images for: Transcriptomic Reprogramming and Genetic Variations Contribute to Western Hemlock Defense and Resistance Against Annosus Root and Butt Rot Disease
Source: Front Plant Sci. 2022 Jun 30;13:908680. doi: 10.3389/fpls.2022.908680 (PMC9279933; doi:10.3389/fpls.2022.908680)

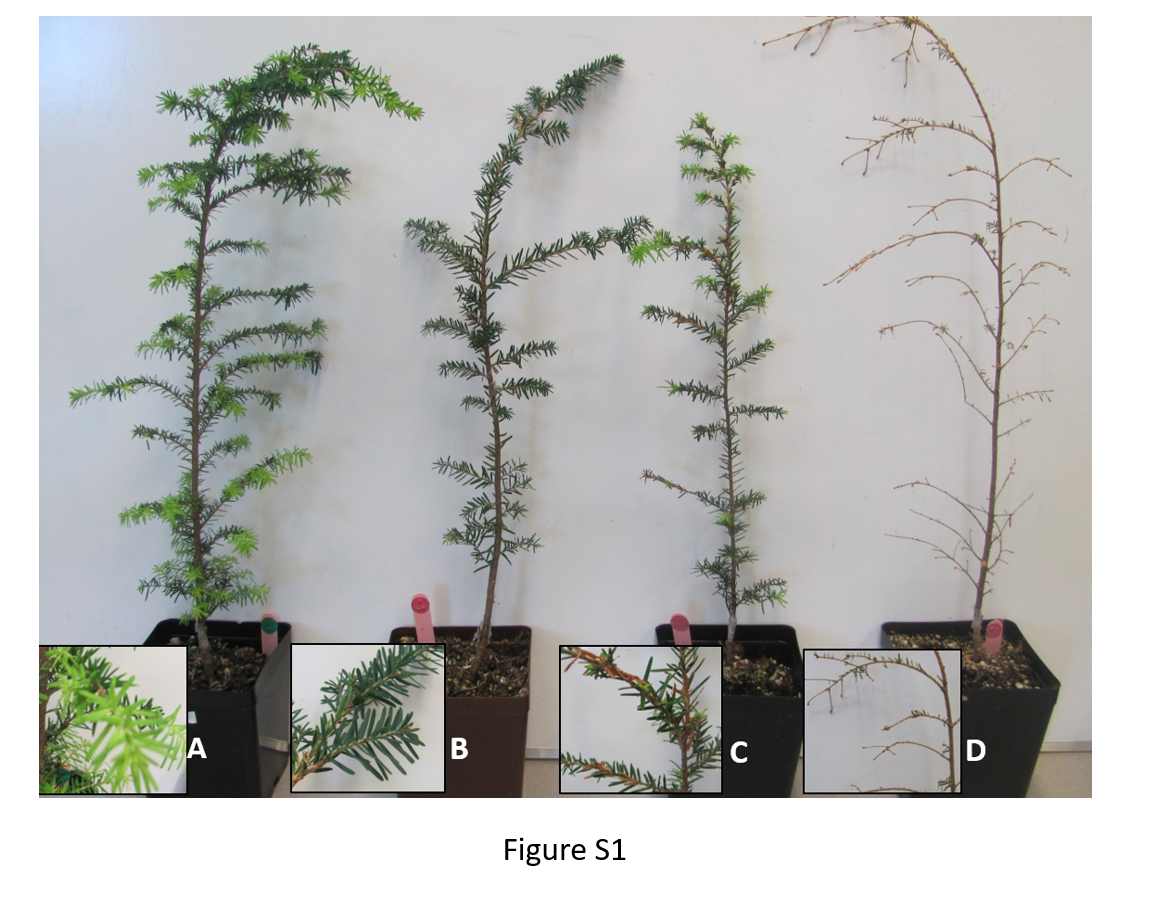

Supplement: Supplementary Figure S1 — Assessment of disease symptoms grouped all seedlings inoculated using Heterobasidion occidentale into four types. (A) Healthy seedlings showed normal growth similar as control seedlings, considered as inoculated but not infected (Uif) type. (B) Infected seedlings showed < 20% of abnormal needles, considered as quantitative resistant (QR) type. (C) Infected seedlings showed 20% ~ 90% of abnormal needles, considered as susceptible (Sus) type. (D) Seedlings died before assessment, and they were not subjected to RNA-seq analysis. [file Image_1.TIF]

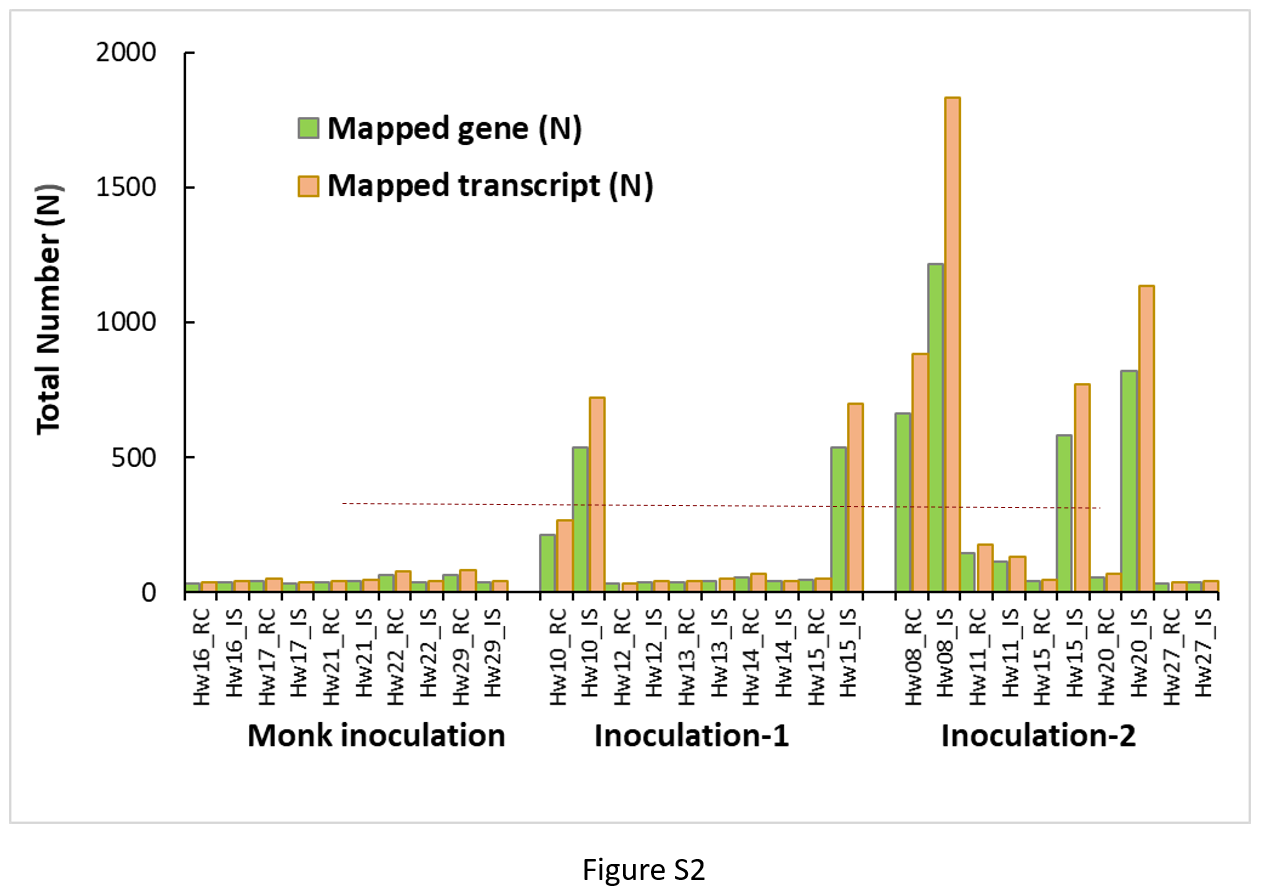

Supplement: Supplementary Figure S2 — Numbers of H. occidentale genes and transcripts detected in western hemlock tissues by RNA-seq analysis. [file Image_2.TIF]

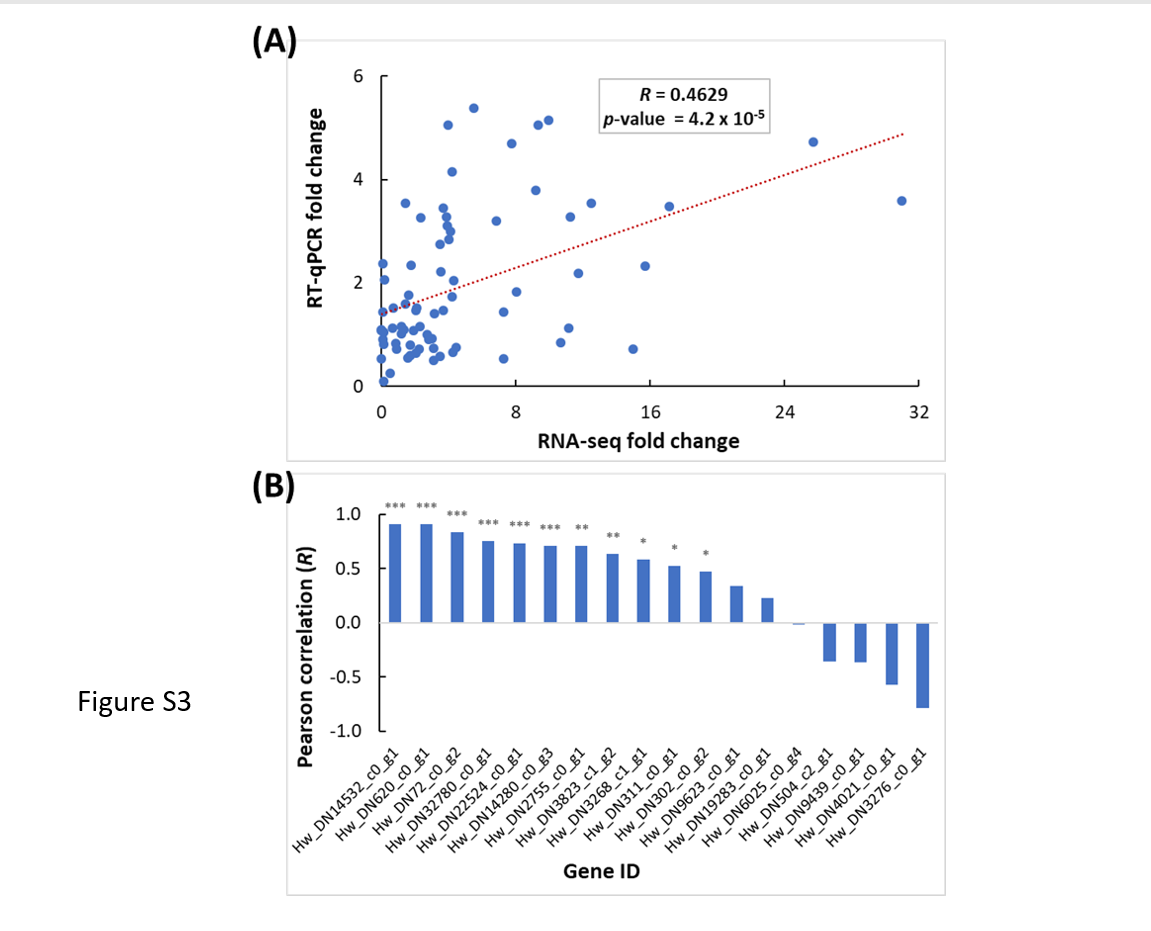

Supplement: Supplementary Figure S3 — Correlation of gene expression levels between measurements by RNA-seq and RT-qPCR. (A) Scatterplot illustrating correlation of fold changes measured between controls (mock inoculation) and susceptible seedlings (Sus), or seedlings with quantitative resistance (QR). Relative transcript expression levels were measured by RNA-Seq-based RPKM and RT-qPCR analyses for 18 selected genes. Fold change of transcript levels were made by comparison of infected seedlings with control seedlings with mock-inoculation. The correlation trend line (red dash) shows a significant positive correlation across all 18 tested genes (Pearson R = 0.46, p = 4.2 × 10^−5^). (B) Correlation of relative quantity (RQ) as measured by RT-qPCR and RPKM as measured by RNA-seq in each tested sample. Transcript expression levels were compared for each of 18 genes. Eleven showed significant positive correlation and classified as concordant genes, while the other seven showed non-significant or negative correlations and classified as non-concordant genes. One to three stars indicate significance of correlations at p < 0.001, 0.01, and 0.05, respectively. [file Image_3.TIF]

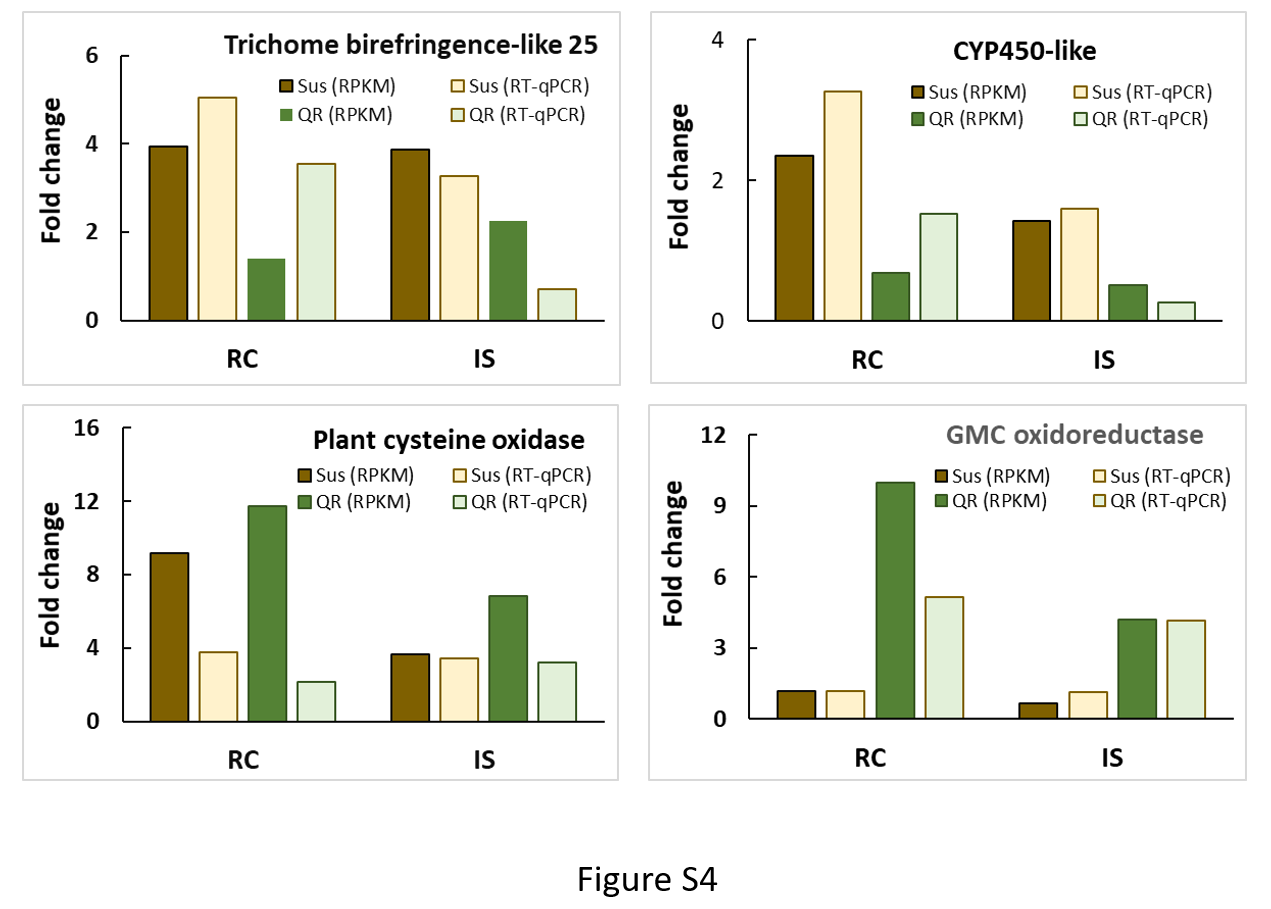

Supplement: Supplementary Figure S4 — Correlation of fold changes for four differentially expressed genes (DEGs) as measured between controls (mock inoculation) and susceptible seedlings (Sus), or seedlings with quantitative resistance (QR). They were annotated as PCY450-like, trichome birefringence-like, GMC oxidoreductase, and plant cysteine oxidase, respectively. [file Image_4.TIF]
